# Sexual size and shape dimorphism, and allometric scaling in the pupal and adult traits of *Eristalis tenax*

**DOI:** 10.1002/ece3.9907

**Published:** 2023-03-15

**Authors:** Jasmina Ludoški, Ljubinka Francuski, Nemanja Gojković, Bojana Matić, Vesna Milankov

**Affiliations:** ^1^ Department of Biology and Ecology, Faculty of Sciences University of Novi Sad Novi Sad Serbia; ^2^ Protix BV Dongen The Netherlands

**Keywords:** allometric intercept, allometric slope, geometric morphometrics, linear morphometrics, ontogenetic allometry, static allometry

## Abstract

The patterns and amount of variation in size, shape, and/or life history traits between females and males are fundamentally important to gain the comprehensive understanding of the evolution of phenotypic diversity. In addition, the covariation of phenotypic traits can significantly contribute to morphological diversification and sexual dimorphism (SD). Using linear and geometric morphometrics, 237 *Eristalis tenax* specimens sampled from five populations were, therefore, comparatively assessed for the variation in sexual size dimorphism (SSD), sexual shape dimorphism (SShD), and life history traits, as well as for trait covariation (ontogenetic and static allometry). Pupal body, adult wing, and body mass traits were analyzed. Female‐biased SSD was observed for pupal length, width, and centroid size, adult wing centroid size, mass, wing loading, and wing area. Conversely, pupal length/width ratio, developmental time, and mass were not found to be sexually dimorphic. Next, wing SShD, but not pupal body SShD was revealed, while allometry was found to be an important “determinant of SD” at the adult stage, with only a minor impact at the pupal stage. By comparing the patterns of covariance (based on allometric slope and intercept) between respective body mass and morphometric traits of pupae and adults, greater variation in allometric slopes was found in adult traits, while static allometries of the two stages significantly differed, as well. Finally, the results indicate that changes in the allometric intercept could be an important source of intraspecific variation and SD in drone fly adults.

## Graphical abstract

The data on the patterns and amount of variation in size, shape, and/or life history traits between females and males (sexual dimorphism, SD) are fundamentally important to gain the comprehensive understanding of the evolution of phenotypic diversity. Female‐biased SSD was observed for pupal length, weight, and centroid size (CS), adult wing CS, mass, and WA, while pupal L/W ratio, PDT, mass, and adult WL were not found to be sexually dimorphic in Eristalis tenax. Shape variation analysis revealed the presence of SShD in wing shape but not in pupal body shape. Allometry was found to be an important determinant of SD at the adult stage, while it had a minor impact at the pupal stage. By comparing the patterns of covariance (based on allometric slope and intercept) for the body mass and morphometric traits of pupae and adults, we found greater variation in allometric slope in adult traits and that the static allometries of the two distinct stages significantly differed.

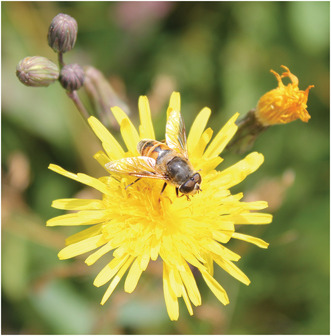

## 1 INTRODUCTION

The study of sexual dimorphism (SD) and evolutionary mechanisms that govern the evolution of phenotypic variation is fundamental for gaining a comprehensive view of evolutionary diversification (see Kraaijeveld et al., 2011 for review). A growing evidence suggests that SD can vary substantially among evolutionary lineages and populations within species (e.g. Sacchi et al., 2015; Tidière et al., 2020; Webb & Freckleton, 2007). Stage‐ and taxon‐specific sexually dimorphic traits (Badyaev, 2002) have also been documented, as well as Rensch's rule that considers the systematic pattern of sex differences in sexual size dimorphism (SSD) within and among species (Rensch, 1959). A variety of evolutionary mechanisms, such as sexual selection, fecundity selection, viability selection, sexually antagonistic selection, and sexual niche segregation, have been proposed as driving forces for the phenotypic divergence between males and females (Blanckenhorn, 2007; Butler et al., 2007; Darwin, 1874; Delph, 2005; Fairbairn et al., 2007; Panhuis et al., 2001). However, the evolution of sexually dimorphic phenotypes, including SSD and sexual shape dimorphism (SShD), might be biomechanically, ontogenetically, genetically, and functionally constrained (Badyaev, 2002; Gidaszewski et al., 2009; Rohner & Blanckenhorn, 2018; Stillwell et al., 2010).

The covariation of phenotypic traits, such as shape and size, could also significantly affect the evolution of SD (Klingenberg, 2010, 2016). The quantification of shape and size, the estimation of their covariance, and the decomposition of shape variation into the components associated and not associated with variation in size can be yielded by using geometric morphometrics (e.g. Benítez et al., 2021; Gidaszewski et al., 2009; Trotta et al., 2011; Vesović et al., 2019). The covariation between the size, morphological, or life‐history traits includes changes that occur during individual ontogenies and those among populations or species (see Klingenberg, 1998, 2016 for review). The research on the scaling relations provided valuable insights into the processes governing morphological diversification (Anzai et al., 2017; Fairbairn, 2005; Griffen et al., 2018; Rohner & Blanckenhorn, 2018; Voje et al., 2014; Voje & Hansen, 2012). For instance, the allometric coefficient (=slope) was suggested to be less variable than the elevation (=intercept) (e.g. Anzai et al., 2017; Bonduriansky, 2007; Egset et al., 2012; Melin et al., 2021), likely reflecting developmental constraint and/or evolutionary conservativism (Bolstad et al., 2015). Moreover, comparative study of allometric pattern showed that most variation among populations exposed to different ecological variables concerns the allometric intercept (Bonduriansky, 2007; Egset et al., 2012; Melin et al., 2021; Pélabon et al., 2014; Voje & Hansen, 2012). Hence, a highly evolvable allometric intercept was supposed to be shaped by natural selection and, thus, has been recognized as an important source of evolutionary potential of population/species (Egset et al., 2012; Pélabon et al., 2014; Melin et al., 2021).

**FIGURE 1:**
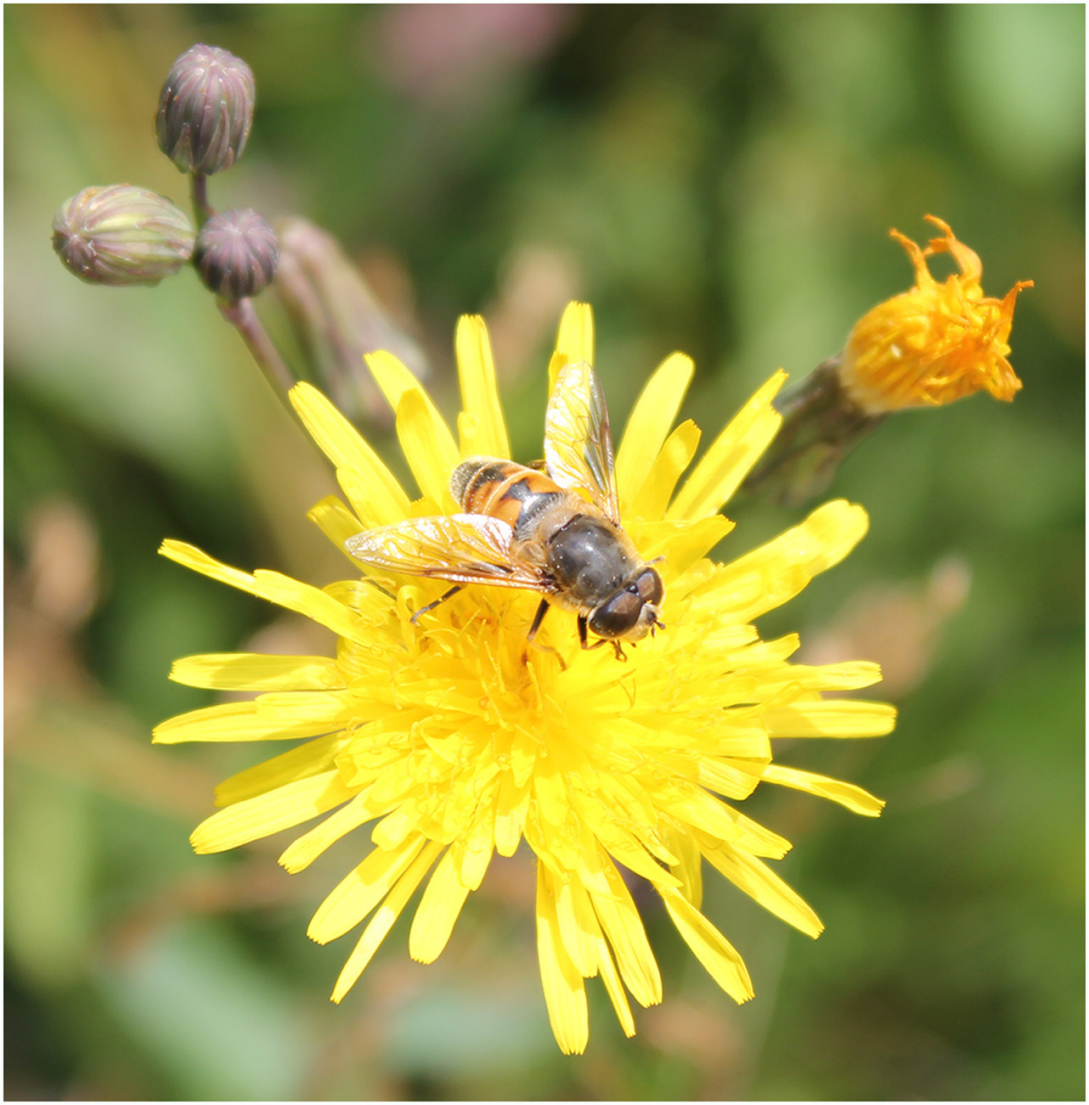
Adult of *Eristalis tenax* (photo by Milica Lukač).

Although SD and phenotypic diversification can contribute substantially to adaptive evolution of holometabolous insects (species that undergo complete metamorphosis), little is still known how microevolution and developmental changes are related in hoverflies, a key group of non‐Hymenopteran pollinators (Lucas et al., 2018). Hence, to bridge this gap, we choose to study drone fly, *Eristalis tenax* (Diptera, Syrphidae) (Figure 1), the species known for its great importance from ecological, conservation, agricultural, economic, and epidemiological perspective (see Francuski et al., 2011; Francuski, Djurakic, Ludoški, et al., 2014; Jauker et al., 2009, 2012; Pérez‐Bañón et al., 2007). Given the multivoltine biology of *E. tenax* (Nicholas et al., 2018), information regarding temporal pattern of genetic and phenotypic variation is of fundamental importance for understanding the capacity of the species to undergo adaptive evolution as well. So far, a significant wing shape variation and abdominal color patterns among temporal samples collected throughout the flight season of drone fly from Fruška Gora Mt was observed (Francuski et al., 2011). However, limited information is available about general pattern of season‐related phenotypes and genotypes of the species, and thus, a comprehensive study of temporal variation in drone fly is strongly needed. Contrary to this, deeper insights into spatial pattern of genetic and phenotypic variation on the local and regional scale were provided. Indeed, landscape genetic studies suggested that populations of this highly migratory species were largely connected (Francuski, Djurakic, et al., 2013; Francuski, Djurakic, Ståhls, & Milankov, 2014; Francuski, Ludoški, & Milankov, 2013; Francuski & Milankov, 2015).

A highly challenging task is understanding the ontogenetic trajectories of sexually dimorphic traits in natural populations of holometabolous species, because the distinct life stages utilize different habitats and resources which promotes different selective pressures on morphological traits (Shingleton et al., 2007; Shingleton & Frankino, 2018). Although ontogenetic mechanisms leading to sex‐related traits still remain insufficiently understood in insects (Sõber et al., 2019), large body of evidence shows that, for instance, SSD appears already during an early larval stage (e.g. Vendl et al., 2018). The drone fly undergoes complete metamorphosis that consists of five developmental phases according to Martín‐Vega et al. (2016) (prepupa, cryptocephalic pupa, phanerocephalic pupa, pharate adult, and imago) while the pupal stage includes the process of intense metabolic, morphological, and behavioral changes (Campoy, Pérez‐Bañón, & Rojo, 2020). To date, the morphometric characterization of hoverfly pre‐adult stages has been mainly based exclusively upon traditional morphometrics using meristic and metrical traits (e.g. Aracil et al., 2022; Pérez‐Bañón et al., 2013; Rotheray & Gilbert, 1999). Contrary to the adult flies, morphological features of premature stage have not been studied for either intersexual or interpopulation differences in *E. tenax* or any other hoverfly species. Actually, to our knowledge, larval and pupal attributes have never been considered as possible features exploitable in the analysis of intraspecific variation, but only rather in describing morphology, providing taxonomic characters, and resolving phylogenetic relationships (e.g. Pérez‐Bañón et al., 2013; Rotheray & Gilbert, 1999).

Considering that a great deal of work including phenotypic plasticity and adaptive diversification of holometabolous insects, including the *Eristalis* species and other hoverflies, remains, in this study, we address the question of the pattern and amount of sex dimorphism in drone fly. We hypothesized that fecundity and natural selection would drive the evolution of SSD and SShD in *E. tenax* individuals. Because fecundity selection tends to favor investment in reproduction by increasing egg production and storage (fecundity‐advantage hypothesis; Darwin, 1874; Stillwell et al., 2010), we expected female‐biased SSD and body mass compared to males. So far, *E. tenax* females were characterized as having greater weight, larger and wider wings, and darker abdomens (Francuski et al., 2011; Francuski, Djurakic, Ludoški, et al., 2014), but smaller eyes, which are separated from each other (Speight, 2020). We also predicted SD in morphological traits related to flight performance (wing shape and wing loading [WL]), as drone fly shows strong sexually dimorphic flight behavior, as males express aggressive behavior associated with territoriality (Wellington & Fitzpatrick, 1981). In addition, both sexes of the species separate activities in space and time (e.g. nighttime shelters, resting‐basking sites, and feeding sites) that may be scattered over a home range of some 500 m^2^ (Wellington & Fitzpatrick, 1981).

Thus, we tested intersexual differences based on the variation in size, shape, and life history traits, as well as their covariation. Specifically, to test whether sexually dimorphic traits are related to ontogenetic stage, we explored both pupal (body length, width, centroid size [CS], shape, and mass, as well as developmental time) and adult (wing CS, shape, loading, and area, as well as body mass) traits. To evaluate if morphological parameters of *E. tenax* scaled differently across traits, populations, and sexes, the particular study aims were to assess: 1. SSD and SShD based on pupal and adult traits; 2. Allometric effect on sexual shape variation; 3. The allometric scaling of morphological covariation by using allometric slope and intercept.

## 2 MATERIALS AND METHODS

### 2.1 The target species

Adult flies are efficient crop pollinators (Howlett & Gee, 2019; Rader et al., 2020) and play a vital role in horticulture, as well as in fruit and vegetable production in greenhouses (Garratt et al., 2014; Jarlan et al., 1997; Takeda & Yanase, 1990). In addition, the rat‐tailed drone fly maggots (the immature stage) play an important ecological role as saprophages in liquid organic matter (Speight, 2020). Indeed, the specimens have been recognized as bio‐indicators and bio‐decomposers, because they live in stagnant, oxygen‐deprived water with a high‐organic content and in manure‐polluted water (see Campoy, Pérez‐Bañón, & Rojo, 2020). The feeding habits of the larval stage promote the possibility of degrading animal waste and transforming it into biomass (Hurtado et al., 2008; Hurtado, 2013).

A lifespan of drone fly lasts about 3 months (Nicholas et al., 2018), with the entire development from pupariation to adult emergence proceeding in 192 ± 3 hr (Campoy, Pérez‐Bañón, & Rojo, 2020). It was shown that at the 30th to 36th hr of the pupal stage (=pharate adult phase), the head, thorax, and abdomen were already differentiated; at the 7th day, the whole body was sclerotized, fully pigmented, and genital structures were well defined, while the imago was fully formed, and wings were still folded at the 8th day (Campoy, Pérez‐Bañón, & Rojo, 2020).

### 2.2 Sample collection

A total of 237 larvae of *Eristalis tenax* were collected a decade ago (in 2011 and 2013) from five localities in three countries: Serbia (Kikinda, Čačak), Montenegro (Orjen Mt, Šasko Lake), and Greece (Litochoro) (Table 1, Appendix S1). Sampling was done during 1‐day visits to pig farms (Table 1). Two temporally distinct samples (2011 and 2013) were taken from Čačak in Serbia, but since preliminary analysis revealed genetic similarity, they were pooled together. Larvae were collected at the end of the larval development (the third instar) from the pig manure and transferred to the Laboratory of Evolutionary Biology at the Department of Biology and Ecology, Faculty of Sciences, University of Novi Sad (Serbia). At the time of sampling, there were numerous adults and larvae on each locality; therefore, it is very likely that the sampled larvae were not the progeny of a single female.

**TABLE 1:** Population origin and sample size of *Eristalis tenax*.

Country	Population	Longitude/latitude	Date	Morphometric analyses
Serbia	Kikinda	20°31′E 45°51′N	21/07/2013	25 (13♀ + 12♂)
	Čačak	20°21′E 43°53′N	24/05/2011, 19/05/2013	82 (38♀ + 44♂)
Montenegro	Orjen Mt	18°31′E 42°28′N	20/06/2011	61(32♀ + 29♂)
	Šasko Lake	19°20′E 41°58′N	25/06/2011	35 (15♀ + 20♂)
Greece	Litochoro	22°28′E 40°06′N	22/05/2011	34 (14♀ + 20♂)
	Total			237 (112♀ + 125♂)

Adults of *E. tenax* are active from early spring to late autumn producing two or three generations each year (Gilbert, 1986). Because drone flies have a lifespan of about 3 months, with time from egg to adult 24–36 days (Nicholas et al., 2018), we assume that our samples of larvae collected in 2011 belong to the same generation (Table 1). Although two subsamples used for additional analyses (please see below) were collected in their larval stage in 2013 (Kikinda and Čačak, Serbia), we consider that allochronic sampling did not influence on the main objective of our study regarding intrapopulation variation in the pattern and amount of SD within and between pupal and adult ontogenetic stages. Still, data on the migration pattern and life cycles of this multivoltine species from the studies localities are lacking.

In the laboratory, larvae were reared in their original medium at room temperature (around 22°C; exposed to indirect sunlight) until they pupated. Because different natural populations might experience different ecological variables during their development (Ottenheim et al., 1995, 1998) and our aim was to observe natural pupal trait variation, larval conditions (manure water content, manure heterogeneity, larval density, etc.) were not standardized. The larval development was finished within 1 or 2 days, after which they were moved to the drier parts of rearing environment where pupation occurred. Pupae were then separated from the manure and individual specimens were placed into separate vials until adult emergence. The adult flies were subsequently stored at −20°C. Species identification, prior to preparation of wing slides and body tissue extracts of adults, was based on the morphological characters of the adults (Hippa et al., 2001). Pupae were photographed and measured before the adult emergence. Morphological analyses of pupae and adults, as well as genetic analyses of adults were performed using the same individuals. Total genomic DNA was extracted from single legs of the specimens, while enzymes were extracted from thorax and head tissues of adults. Remains of specimens used for the geometric morphometric analyses, allozyme study and DNA extraction are deposited at the Laboratory of Evolutionary Biology, UNSPMF.

To infer whether population connectivity influenced the spatial distribution of intraspecific phenotypic diversity, we analyzed the genetic homogeneity of the samples originating from different geographical regions by using three independent molecular markers of mitochondrial (cytochrome *c* oxidase subunit I gene, *COI* mtDNA) and nuclear (five allozyme loci and internal transcribed spacer 2 locus of the ribosomal DNA cluster, *ITS2* rDNA) DNA. In total, 71 individuals were used for the assessment of allozyme loci variability and 12 individuals for the inspection of *COI* mtDNA (655 bp) and *ITS2* rDNA (437–439 bp) polymorphism (Appendix S2). The sample included data from both the previous studies of *E. tenax* molecular variability (allozyme loci and partial *COI* mtDNA sample) (Francuski, Djurakic, Ståhls, & Milankov, 2014; Francuski & Milankov, 2015) and the newly obtained sequences (the remaining *COI* mtDNA and *ITS2* rDNA sequences) (Appendix S3). The details regarding genetic analyses are given in Appendix S4.

### 2.3 Morphometric analysis

To analyze phenotypic variation of 237 individuals at both pupal and adult developmental stage, two morphometric approaches were applied: linear (traditional) and landmark‐based geometric morphometrics. The digital images of pupal dorsal and ventral views were taken using a full frame 3.3 MP digital camera Leica DFC320, connected to a stereomicroscope Leica MZ12.5 under the same magnification (0.8× objective). Images were acquired in RGB 24‐bit color mode with 2088 × 1550 pixel resolution and saved in jpg format. As the length of the pupal period varies from 5 to 10 days under laboratory conditions (Pérez‐Bañón et al., 2013: 8–10 days; Campoy, Pérez‐Bañón, & Rojo, 2020: 5–9 days), all pupae were 2‐day old at the moment of capturing images, meaning that they were at the same point of development. Dorsal image views were used for both linear and geometric morphometric analysis.

Using the same equipment and settings, digital images of *E. tenax* wings were collected. Prior to capturing wing images, the right wings of all specimens were removed and mounted in Hoyer's medium between microscope slides.

In addition, for the subsample that included 25 and 41 individuals collected in their larval stage at Kikinda and Čačak (Serbia), respectively, the weight of individuals at both pupal and adult life stages was measured using the digital weighing scale Adventurer AR 1530 (Ohaus) to the nearest 0.001 g. Pupae were weighted when they were 3‐day old (at approximately the same time they were imaged for morphometric analysis), while adults were 1‐ to 2‐day old (i.e. when their wings fully hardened).

#### 2.3.1 Linear morphometrics

**FIGURE 2:**
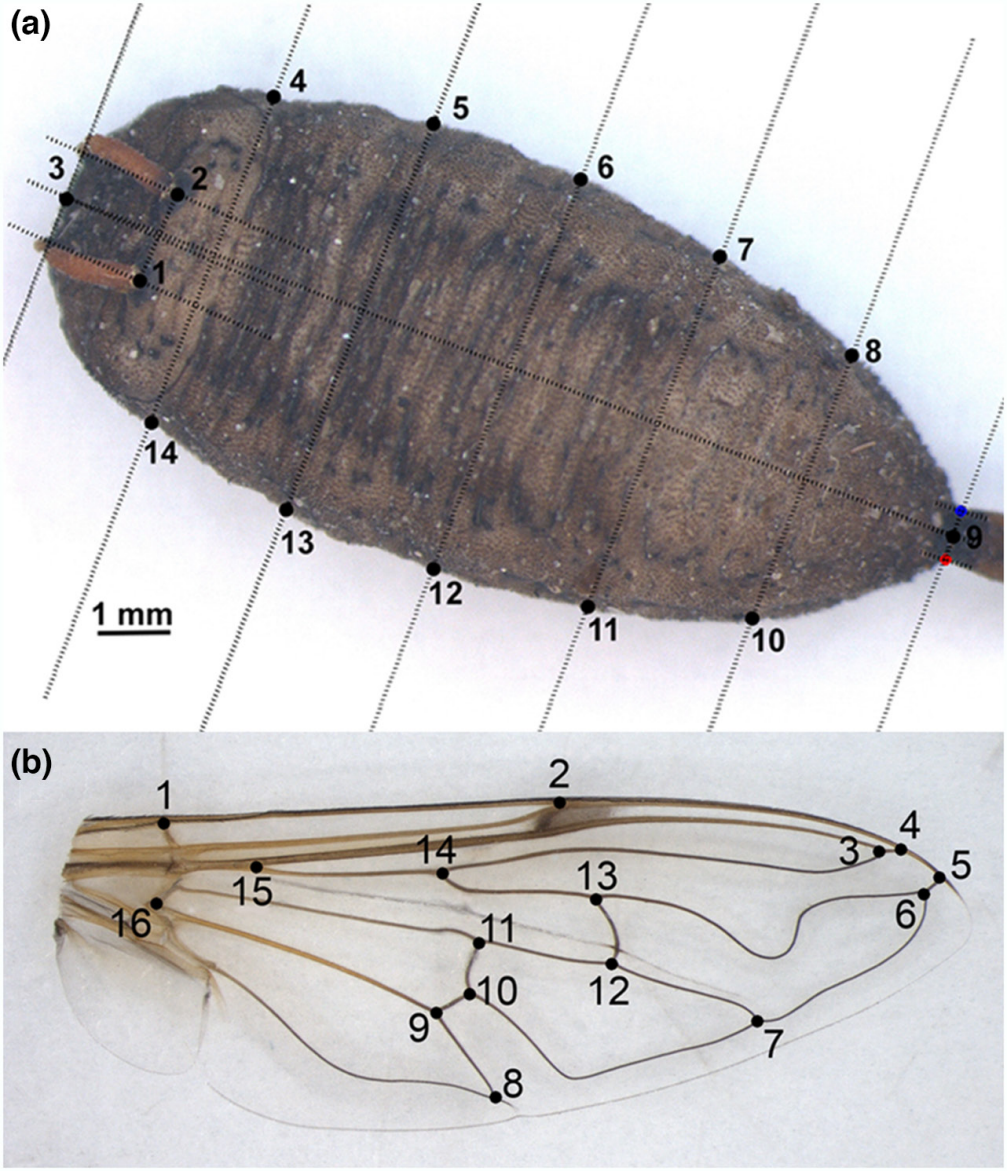
(a) Position of landmarks (1, 2) and semilandmarks (3–14) selected for geometric morphometric analysis of *Eristalis tenax* pupae. For linear morphometric analysis, pupal length and width were measured as distance from point 3 to 9 and from 6 to 12, respectively. (b) Position of 16 landmarks selected for wing geometric morphometric analysis of *Eristalis tenax* flies.

For each pupa, two straight line distances representing the length (L) and width (W) of pupal body were measured to the nearest 0.01 mm (Figure 2a) using the measure mode in tpsDig2 ver. 2.31 software (Rohlf, 2017a). Length was measured along the middle line of pupal body from the top of the head to the base of the tail (distance between landmarks 3 and 9 on Figure 2a), while W was measured at the middle of body (distance between landmarks 6 and 12 on Figure 2a). The L/W index was also calculated, representing the ratio of L to W measurements. Besides, the measures collected for overall sample (L, W), for the subsample of 66 individuals from Kikinda (13 female and 12 male) and Čačak (17 female and 24 male) for which pupal and adult mass was measured (see above), additional adult traits wing area (WA) and WL were also obtained. The WA (in mm^2^) was measured with the curve draw mode in tpsDig2 ver. 2.31 software (Rohlf, 2017a), as the area of enclosed region bounded by a curved line along the outline of the wing from landmark 1 to alula incision (the end point was connected to the starting point so that the curve is closed). Then, WL (in mg/mm^2^) was calculated as: WL = body mass/(2 × right WA).

#### 2.3.2 Geometric morphometrics

Coordinates of two landmarks and 12 semilandmarks were digitized on each pupa image, while16 landmarks were placed on every wing image using tpsDig2 ver. 2.31 (Rohlf, 2017a). For pupae, landmarks (points 1 and 2) were placed at the base of antennae (horn‐like processes on the dorsal side of head), while semilandmarks (points 3–14) were placed along the edges of the pupal body (Figure 2a). Landmarks represent morphologically well‐defined, biologically homologous points that can be recognized as the same point in all individuals in the study (Zelditch et al., 2012). When landmarks are scarce, the morphological information can be captured using additional points, so‐called semilandmarks, that are not discrete anatomical loci but are placed relative to one another following some consistent rule (MacLeod, 2013; Zelditch et al., 2012). In this paper, the positions of semilandmarks were defined using a “comb” of equally spaced lines produced with MakeFan8 (Sheets, 2014). For wing geometric morphometrics, landmarks were positioned at vein intersections or terminations (Figure 2b). Each wing was digitized two times, and both replicate measurements were subjected to a Procrustes ANOVA in order to evaluate a measurement error due to the digitizing process (Klingenberg et al., 2002). The results revealed that for both wing size and shape, the Procrustes mean squares for individual variation substantially exceeded measurement error. Therefore, for wing shape analysis, we performed the replicate measurements of each specimen which were averaged.

Raw landmark coordinates were superimposed using a generalized Procrustes analysis in tpsRelw ver. 1.69 software (Rohlf, 2017b) with a minimum Procrustes distance criterion for alignment of semilandmarks (Bookstein, 1997; Perez et al., 2006). As a result, CS and shape information (Procrustes coordinates) were extracted from the landmark data. Being bilaterally symmetrical, the pupal body has object symmetry; therefore, the symmetric component of total shape variation (among‐individual variation in the average of right and left side landmark configurations) was used for shape analyses (Klingenberg et al., 2002).

#### 2.3.3 Statistical analysis

All morphometric and statistical analyses were done using MorphoJ 1.07a (Klingenberg, 2011), Statistica 14 (TIBCO Software Inc., 2020), and PAST 4.10 (Hammer et al., 2001).

To estimate SSD, two‐way multivariate analysis of variance (MANOVA) with sex and population (five populations) as main effects was performed on pupal L, W, L/W, CS, and wing CS measurements (pupal and adult body mass, WA, and WL were not included, as they were measured only for the subsample). Wing measurement was used as a surrogate measure of body size, as wing size in insects is roughly correlated with their body size (Grimaldi & Engel, 2005). Since MANOVA revealed significant effects (see Results), two‐way ANOVA for each measurement was done separately. Within each population, the difference in mean trait values between sexes was tested with *t*‐test. In addition, measurements (pupal and adult body mass, WA, and WL) obtained for the subsample (Kikinda and Čačak) were also tested for intersexual differences using *t*‐test. Finally, for all measurements, SD index (SDI; after Lovich & Gibbons, 1992) was calculated as SDI = (mean size of larger sex/mean size of smaller sex)−1.

To explore the pattern of interpopulation SSD and the existence of Rensch's rule, a major axis (MA) model of linear regression was performed; log male size was regressed on log female size. A slope greater than 1 (hyperallometry) indicates that evolutionary divergence caused by sexual selection on males has exceeded the one caused by reproductive selection on females (Abouheif & Fairbairn, 1997; Blanckenhorn et al., 2007). The statistical significance of MA slope (i.e. deviation from isometry, MA slope = 1) was tested using the bootstrapped 95% confidence intervals (1999 replicates). For comparing regression slopes, chi^2^ test was employed. Sex differences in allometric slopes and intercepts were assessed by performing linear regression of different pupal (L, W, L/W, CS) and adult (WA, WL, CS) log traits on log body mass (pupal and adult, respectively). These included both ontogenetic (shape changes in the same individual through developmental time; herewith pupa and adult stages) and static (individuals at the same developmental stage within sex/population/species) allometry. The allometric patterns of morphological covariation are described by allometric slope (*b*) and intercept (*a*), based on the equation *Y* = *aX* ^*b*^, where *Y* and *X* represent trait size and body size, respectively (Huxley, 1924, 1932).

To explore SShD, a discriminant analysis (DA) on both pupal body and adult wing shape variables was done. The amount of SShD was quantified in the units of Procrustes distance between sexes. Shape changes associated with the discriminant variable were graphically visualized with deformation grids. The multivariate regression of Procrustes coordinates against CS on pooled within‐group variation (pooled by sex) was performed in order to evaluate the allometric effect (the relationship between size and shape). A permutation test with 10,000 iterations was run to estimate the statistical significance of the allometry. The homogeneity of sex‐specific allometric slopes was tested by the multivariate analysis of covariance (MANCOVA) with shape variables as dependent variables, population and sex as categorical factors, and CS as a covariate. In addition, the predicted values and residuals from regression were also subjected to DA to obtain non‐allometric and allometric components of total SShD, respectively.

## 3 RESULTS

Genetic homogeneity of the *E. tenax* populations was supported by the retrieval of no variation at *COI* mtDNA and *ITS2* rDNA loci, as well as by the lack of population structure (based on both non‐spatial and spatial analyses of allozyme data) (Results are given in Appendix S4).

### 3.1 Sexual dimorphism

#### 3.1.1 Pupal developmental time

In all *E. tenax* samples, pupal developmental time (PDT) lasted from 7 to 8 days. During eclosion, the emergence of females and males highly overlapped. This simultaneous emergence of both sexes indicated that there was a lack of SD in PDT in the study species.

#### 3.1.2 Sexual size dimorphism

Two‐way MANOVA conducted on the dataset containing five pupal and adult measurements (L, W, L/W, pupa CS, and adult CS) collected for the 237 individuals of five populations yielded significant effects of population (Wilks' Λ = 0.427; *F*_(20, 741)_ = 10.827; *p* < .001) and sex (Wilks' Λ = 0.335; *F*_(5, 223)_ = 88.513; *p* < .001), but not of population by sex interaction (Wilks' Λ = 0.925; *F*_(20, 741)_ = 0.876; *p* = .62), which indicates consistent pattern of SSD across populations. Separate ANOVAs (performed for each of the five considered variables) revealed a significant difference between sexes in pupal body L (*F* = 7.567; *p* < .001), W (*F* = 7.728; *p* < .001), and CS (*F*_(1, 227)_ = 8.754; *p* < .001) and adult wing CS (*F*_(1,227)_ = 188.033, *p* < .001), but not for L/W index (*F* = 0.312; *p* = .58) (Table 2). As for MANOVA, the interaction of factors (population × sex) was not significant (*p* > .05) for any measurement, indicating the lack of SSD divergence among populations (Table 2).

**TABLE 2:** The results of ANOVA on the size measures of *Eristalis tenax* pupae and adults.

Effect	SS	*df*	MS	*F*	*p*
L					
Population	71.3520	4	17.8380	28.2372	<.001
Sex	4.7802	1	4.7802	7.567	<.001
Pop × sex	2.1900	4	0.5475	0.867	.4847
Error	143.4032	227	0.6317		
W					
Population	8.1087	4	2.0272	12.738	<.001
Sex	1.2299	1	1.2299	7.7283	<.001
Pop × sex	0.5438	4	0.1359	0.854	.4923
Error	36.1257	227	0.1591		
L/W					
Population	0.5077	4	0.1269	7.736	<.001
Sex	0.0051	1	0.0051	0.312	.5772
Pop × sex	0.0212	4	0.0053	0.323	.8625
Error	3.7242	227	0.0164		
Pupa CS					
Population	1,883,156.9045	4	470,789.2261	25.947	<.001
Sex	158,839.2148	1	158,839.2148	8.754	<.001
Pop × sex	69,247.4803	4	17,311.8701	0.954	.4336
Error	4,118,744.7710	227	18,144.2501		
Adult CS					
Population	240,155.7260	4	60,038.9315	11.725	<.001
Sex	962,845.6489	1	962,845.6489	188.033	<.001
Pop × sex	18,428.8386	4	4607.2096	0.900	.4649
Error	1,162,379.5023	227	5120.6145		

*Note*: Pupal body traits: length (L), width (W), length/width ratio (L/W), and pupal body centroid size (pupa CS); adult trait: wing centroid size (adult CS).

**FIGURE 3:**
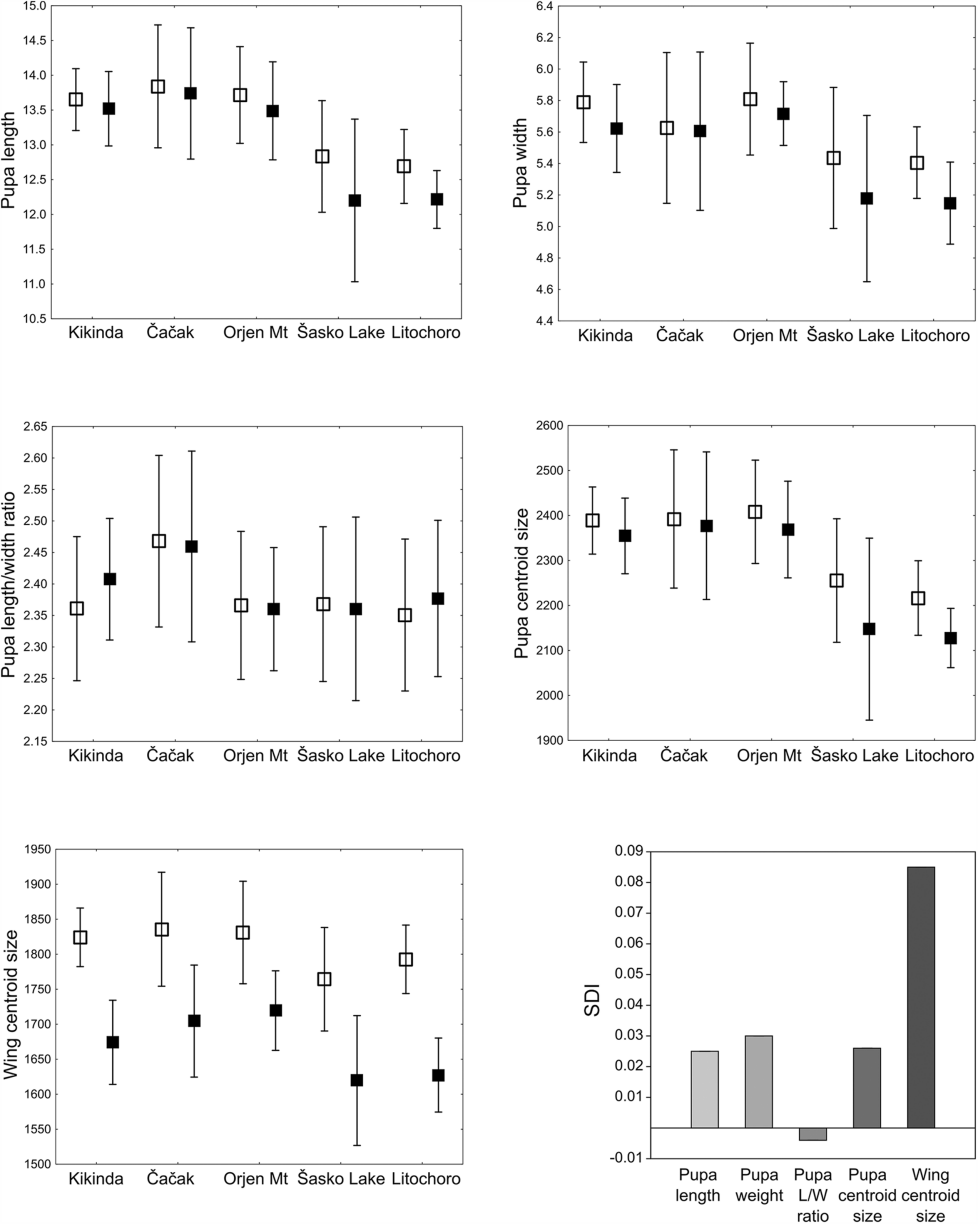
Trait mean size across populations and sexes and SDI of *Eristalis tenax* pupae and adults. Whiskers represent the standard deviation of the mean. Females ‐ empty squares; males ‐ filled squares.

The mean values of pupal and adult morphometric traits were higher in females (with a few exceptions for L/W index), revealing SDI values which ranged from 0.000 to 0.052 for pupal traits and from 0.065 to 0.102 for adult CS (Figure 3, Appendix S7). Pupal L, W, and CS significantly differed between sexes (*t*‐test *p* < .01) only in Litochoro population, while SD in adult wing CS values was statistically significant (*t*‐test *p* < .001) within each population (Appendix S7).

When the traits measured for *E. tenax* subsample (66 individuals from Kikinda and Čačak populations) were analyzed, and female‐biased SSD (FBSSD) was found for both pupal and adult body mass, although intersexual differences were statistically significant only between adults in Čačak population (*t*‐test *p* < .05) (Table 3). Besides that, female adults had also significantly higher WA value (*t*‐test *p* < .001) than males in both populations, while WL values were higher in males, although being significant (*t*‐test *p* < .05) only in Kikinda population (Table 3).

**TABLE 3:** Descriptive statistics (mean ± SD) for body mass, wing area (WA), and wing loading (WL) of *Eristalis tenax* subsample comprising Kikinda (13 females and 12 males) and Čačak (17 females and 24 males) populations.

	Female	Male	*t*‐test	SDI
	Mean ± SD	Mean ± SD		
Pupal body mass				
Kikinda	166.38 ± 16.47	158.83 ± 18.03	ns	0.048
Čačak	194.06 ± 22.55	182.17 ± 23.70	ns	0.065
Adult body mass				
Kikinda	91.67 ± 11.28	86.25 ± 10.00	ns	0.063
Čačak	107.59 ± 17.48	94.00 ± 16.23	*	0.145
WA				
Kikinda	31.14 ± 1.50	26.78 ± 1.88	***	0.163
Čačak	34.19 ± 1.31	29.18 ± 1.86	***	0.172
WL				
Kikinda	1.47 ± 0.13	1.61 ± 0.15	*	−0.087
Čačak	1.57 ± 0.24	1.61 ± 0.25	ns	−0.025

*Note*: Differences between sexes were tested with *t*‐test (ns, not significant, *p < .05, ***p < .001). Sexual dimorphism index (SDI) was calculated after Lovich and Gibbons (1992).

#### 3.1.3 Total sexual shape dimorphism

Regarding total SShD, DA on pupal body shape variables did not find statistically significant differences between sexes for any of the populations, whereas wing SShD was present (Table 4). More precisely, the amount of total wing SShD (quantified as Procrustes distance between the mean wing shape of the males and females) differed among populations. Kikinda was the most sexually dimorphic population, while Šasko Lake displayed the least amount of shape dimorphism (Table 4). Contrary to the similarity in the pattern of SSD, the magnitude of total SShD quantified on pupal body shape and adult wing shape differed between two developmental stages, with Procrustes distances between sexes being at least two to three times greater in adults (Table 4).

**TABLE 4:** Differences between the mean shapes of *Eristalis tenax* females and males revealed by discriminant analysis (DA).

	Total SShD	Non‐allometric SShD	Allometric SShD
Pupal body shape			
Kikinda	0.0050^ns^	0.0053^ns^	0.0011^ns^
Čačak	0.0042^ns^	0.0044^ns^	0.0004^ns^
Orjen Mt	0.0021^ns^	0.0023^ns^	0.0014^ns^
Šasko Lake	0.0041^ns^	0.0033^ns^	0.0012^ns^
Litochoro	0.0045^ns^	0.0089^ns^	0.0056**
Wing shape			
Kikinda	0.0161**	0.0164***	0.0081***
Čačak	0.0128***	0.0157***	0.0083***
Orjen Mt	0.0111***	0.0147***	0.0069***
Šasko Lake	0.0101**	0.0162***	0.0140***
Litochoro	0.0134***	0.0173***	0.0136***

*Note*: The amount of sexual shape dimorphism (SShD) was quantified in the units of Procrustes distance. To obtain the non‐allometric and allometric component of total SShD, DA was performed on residuals and predicted values, respectively, from the multivariate regression of shape on centroid size. **p < .01; ***p < .001.

Abbreviation: ns, not significant.

A wireframe graph illustrating pupal body shape variation revealed landmark pairs 1–2, 5–13, and 7–11 as those with the greatest contribution to variability (Appendix S8a). The visualization of wing shape changes showed that differences between sexes across populations is predominantly due to the displacement of landmarks 8 and 14, followed by landmarks 2, 7, and 13 (Appendix S8b).

#### 3.1.4 Allometric effect on sexual shape variation

The MANCOVA analysis found a non‐significant allometric effect of CS on pupal body shape, but a significant one on wing shape variation. In addition, there were no differences among populations and sexes in allometric pattern (non‐significant Sex × CS and Pop × Sex × CS interactions) (Table 5). The multivariate regression of pupa body shape on pupa body CS showed that allometry explained between 0.67% and 4.77% of total shape variation (*p* < .05), being significant only in Čačak (3.60%, *p* < .05) (Appendix S9). The multivariate regression of adult wing shape on wing CS showed that allometry explained between 1.67% and 22.37% of total shape variation, being significant in Čačak (7.02%, *p* < .001), Orjen Mt (5.02%, *p* < .01), and Šasko Lake (22.37%, *p* < .001) populations (Appendix S9).

**TABLE 5:** MANCOVA test with shape variables as dependent variables, centroid size (CS) as continuous, and sex and population as categorical variables.

	Pupal body shape			Wing shape		
Effect	Wilk's Λ	*df*	*F*	Wilk's Λ	*df*	*F*
Population	0.640	48, 796	2.037***	0.4093	112, 757	1.705***
Sex	0.940	12, 206	1.096^ns^	0.855	28, 190	1.154^ns^
CS	0.917	12, 206	1.546^ns^	0.752	28, 190	2.237***
Pop × sex	0.848	48, 796	0.726^ns^	0.535	112, 757	1.154^ns^
Pop × CS	0.662	48, 796	1.874***	0.412	112, 757	1.692***
Sex × CS	0.940	12, 206	1.105^ns^	0.858	28, 190	1.122^ns^
Pop × sex × CS	0.847	48, 796	0.729^ns^	0.534	112, 757	1.141^ns^

Abbreviation: ns, not significant.

*** *p* < .001.

Considering the two components (non‐allometric and allometric) of total shape variation separately, the DA on pupal body variables revealed significant difference between sexes only for the allometric component in Litochoro, while the wing shape analysis of both components discovered statistically significant distances between sexes for each population (Table 4). The pattern of SD in both pupal body shape (Appendix S8a) and adult wing shape (Appendix S8b) did not notably change when the shape variables were corrected for the allometric effect.

#### 3.1.5 Allometric scaling of morphological traits

**FIGURE 4:**
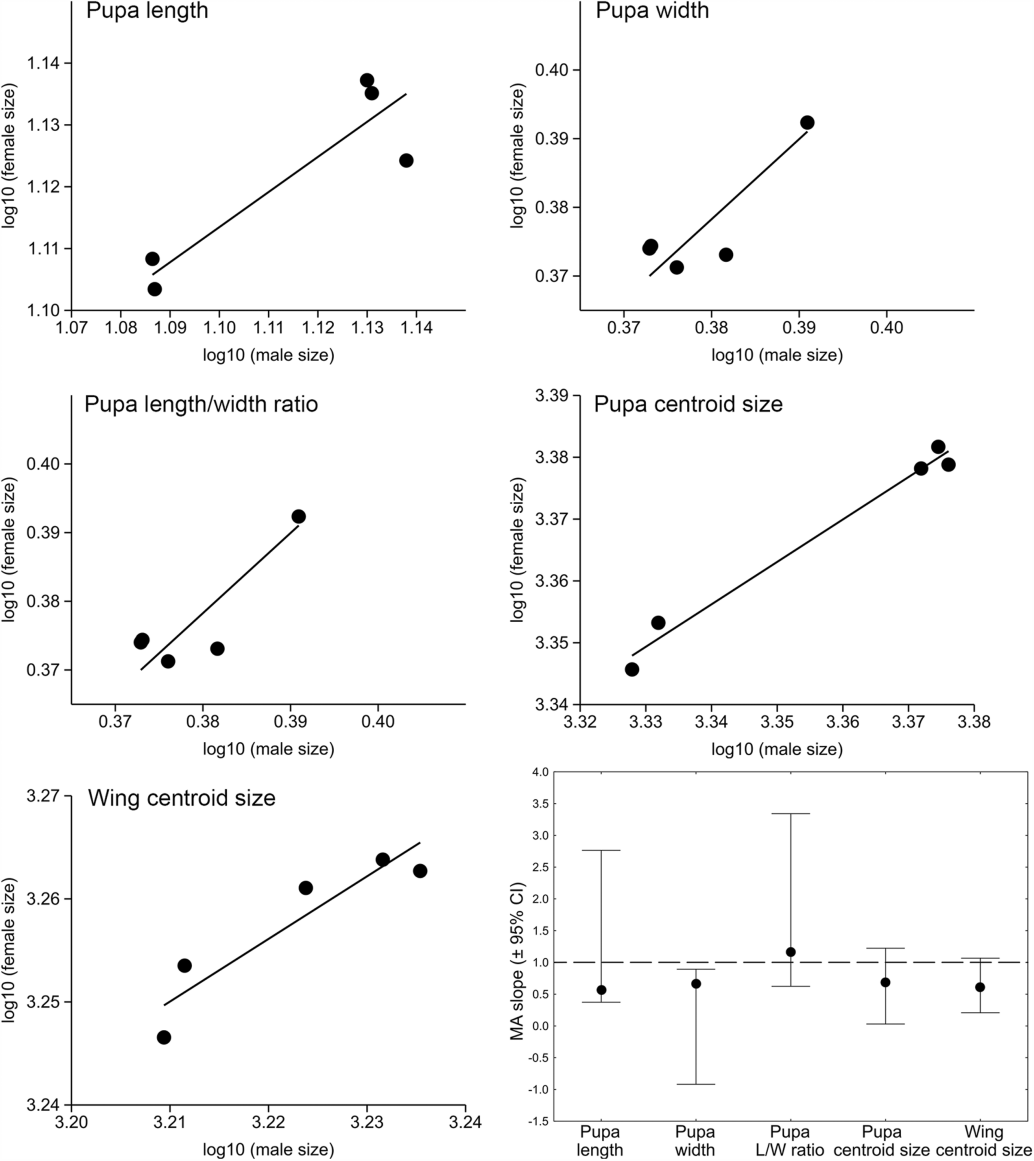
The major axis linear regression of female and male log‐transformed mean sizes for the pupal and adult morphometric traits of *Eristalis tenax*.

**FIGURE 5:**
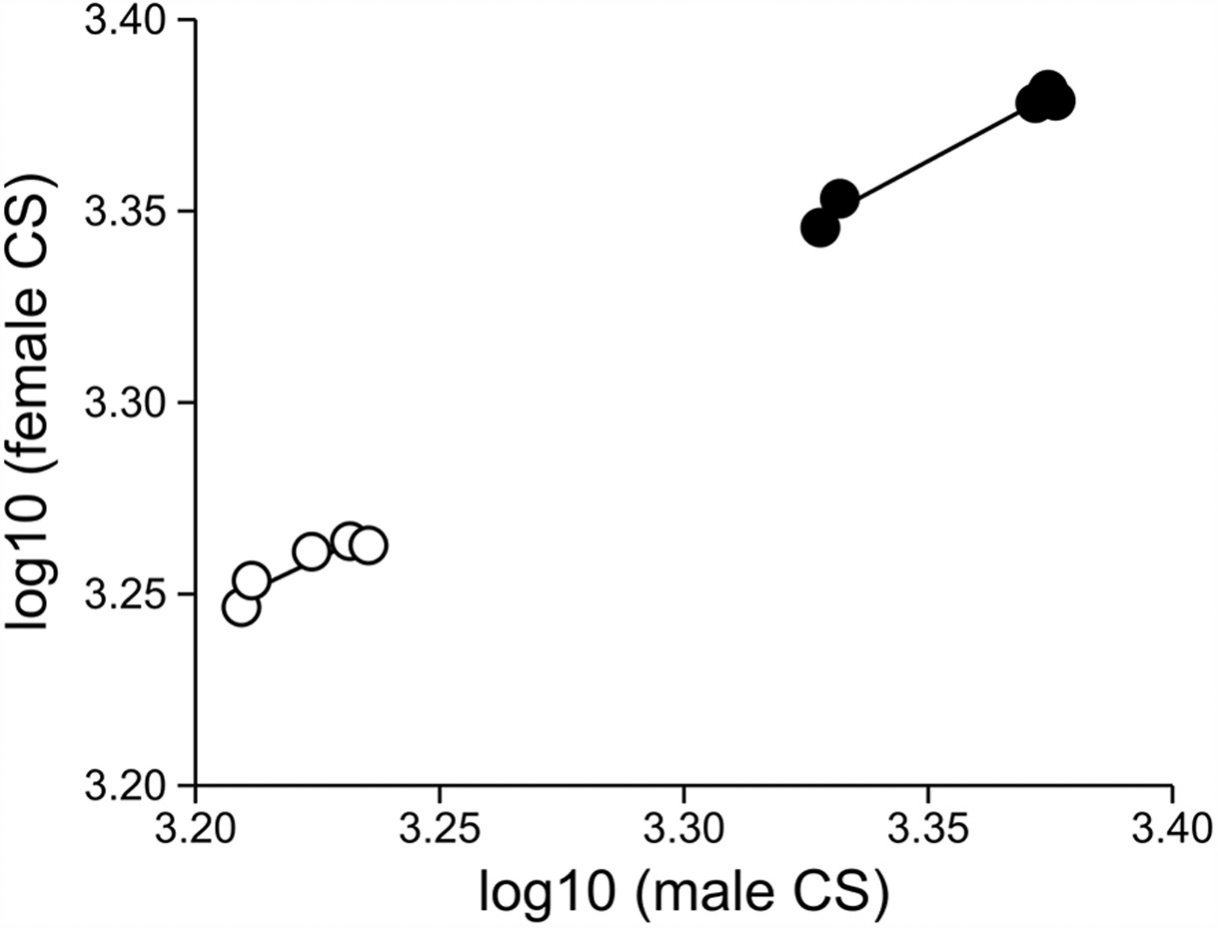
Allometric relationship between the log‐transformed values of male versus female centroid size at the pupal (filled circle; MA slope = 0.686, *a* = 1.064) and adult (empty circle; MA slope = 0.608, *a* = 1.300) developmental stages of *Eristalis tenax*.

The major axis regression of male and female log‐transformed mean size values revealed regression slopes lower than 1 for W (MA slope = 0.670; 95% CI −0.919–0.892), whereas the isometric relationship was found for L (MA slope = 0.568; 95% CI 0.373–2.764), L/W ratio (MA slope = 1.167; 95% CI 0.624–3.341), pupal body CS (MA slope = 0.686; 95% CI 0.030–1.223), and wing CS (MA slope = 0.608; 95% CI 0.208–1.067). However, the comparison of these five regressions showed no significant difference in allometric slopes (chi^2^ = 1.837, *p* = .76) (Figure 4, Appendix S7). In addition, the comparison of MA regressions of male and female log‐transformed mean CS values at two developmental stages showed no significant difference in slopes (chi^2^ = 0.2013, *p* = .65; Figure 5).

**FIGURE 6:**
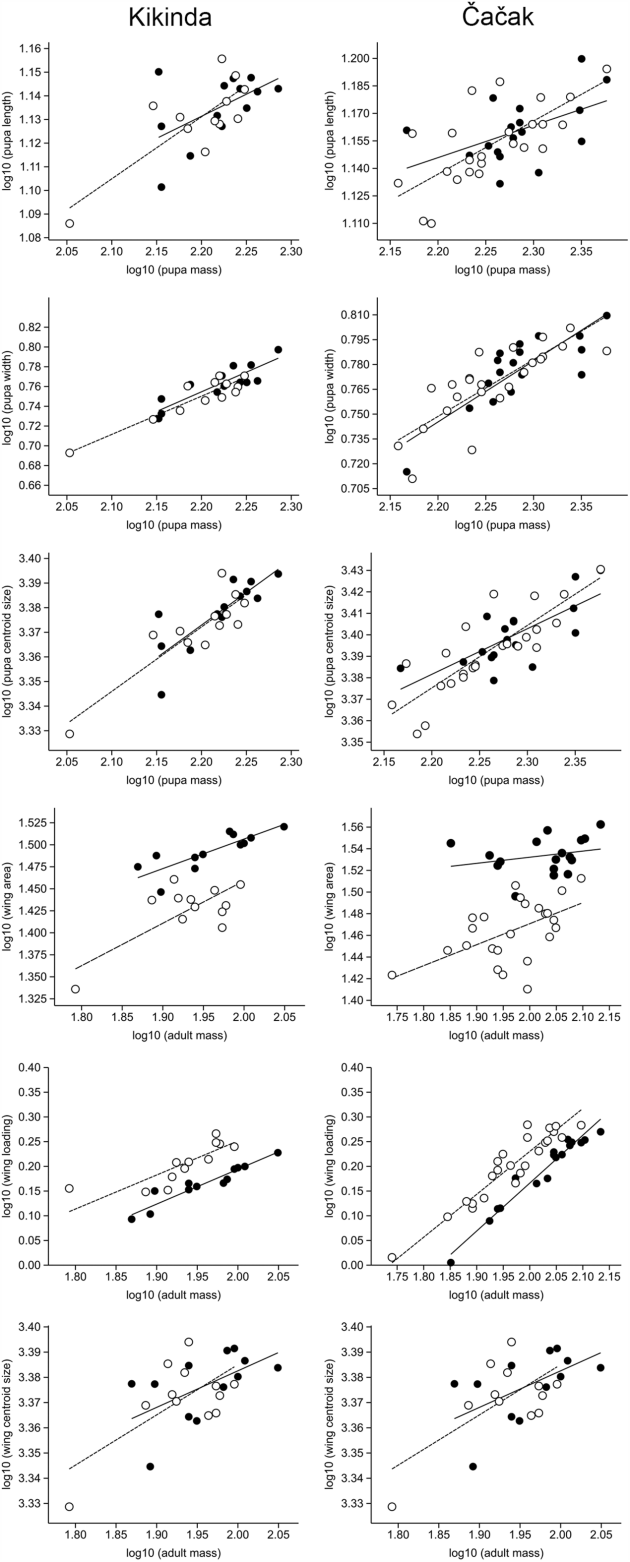
The major axis linear regression of pupal body length, width, and centroid size on pupal body mass, wing area, wing loading, and wing centroid size on adult body mass across the sexes of *Eristalis tenax*. Females: black circle; males: white circle.

Regarding the traits measured only for the Kikinda and Čačak subsample, the major axis regression of log‐transformed body mass against pupal and adult size measures across sexes revealed the positive relationship in both females and males. Furthermore, chi^2^ test showed that female and male regression slopes did not differ significantly (*p* > .05) (Figure 6, Appendix S10). MA slopes obtained for adult traits showed a larger range of variation in both sexes (females: 0.056–0.969; males: 0.141–0.868) than pupal traits (females: 0.176–0.398; males: 0.262–0.387) (Appendix S10). In addition, except for W and WL, allometric intercept value for the remaining traits was higher in females and also varied greatly for both pupal (females: −0.120–2.914; males: −0.101–2.796) and adult traits (females: −1.773–3.161; males: −1.507–3.144) (Appendix S10).

## 4 DISCUSSION

### 4.1 Genetic connectedness of the drone fly samples

Regarding the general objective of the current study, the variation in the patterns and amount of SD within and between pupal and adult ontogenetic stages of drone fly was comprehensively analyzed. In this study, molecular markers of mtDNA and nDNA were used to test genetic homogeneity of the analyzed populations. The lack of variation at *COI* mtDNA and *ITS2* rDNA sequences, as well as population genetic structure analyses based on allozyme nDNA genes, suggested that a genetically homogenous sample was used in our study. This is congruent with the previously described pattern of considerable gene flow among the geographically distinct samples of this species, capable of long‐distance dispersal in a spatially heterogeneous environment both at the local (Francuski, Ludoški, & Milankov, 2013) and broader geographical scale (Francuski, Djurakic, et al., 2013; Francuski, Djurakic, Ståhls, & Milankov, 2014; Francuski & Milankov, 2015). Hence, genetic connectedness of the analyzed populations likely has not influenced the patterns and amount of intersexual and interpopulation phenotypic variation observed in our study.

### 4.2 The consistent pattern of female‐biased sexual size dimorphism in the pupal and adult traits of drone fly

In the present study, the hypothesis of sexual size and sexual shape dimorphism was tested by using pupal and adult traits. With respect to our first goal, the consistent pattern of FBSSD was observed for pupal body length (L), pupal body width (W), and pupal body CS, as well as adult WA and wing CS, while pupal L/W ratio and pupal body mass were not found to be sexually dimorphic traits. However, negligible interpopulation differences in SD were detected in adult body mass and adult WL. Such consistent pattern of SD was supported by the results of SDI analyses, where traits with significant SSD at both pupal (L, W, CS) and adult (body mass, WA, and CS) stage had greater SD indexes. Not only were females characterized by the higher mean values of size measures (L, W, body CS, WA, wing CS) but also by the greater body mass than males at both stages.

Female‐biased SSD observed in our study is complementary to the findings of the studies of drone fly populations (Francuski et al., 2011; Francuski, Ludoški, & Milankov, 2013) and several eristaline species (*Eristalis arbustorum*: Francuski et al., 2020; *E. interrupta*: Francuski, unpublished data), *Merodon* species (*M. albifrons*: Milankov et al., 2013; *M. avidus*: Milankov et al., 2009; *M. testaceus*: Ludoški, unpublished data), *Syrphus ribesii* (Gilbert, 1985), and *Sphaerophoria scripta* (Gojković et al., 2020). Hence, our results essentially confirmed the findings of the previous studies in insects (e.g. Le Roy et al., 2019; Stillwell et al., 2010; Teder, 2014) which showed that females were larger and heavier than males probably because of the impact of natural selection. Therefore, our findings support the fecundity‐advantage hypothesis (Darwin, 1874; Stillwell et al., 2010) that proposes fecundity selection as the main factor promoting greater abdominal mass and larger body size in females (see Wickman, 1992).

### 4.3 Lack of sexual dimorphism in drone fly pupal developmental time

We observed that PDT highly overlapped between sexes and populations, and as such, the lack of sex differences in PDT is consistent with the published findings on the pupal development of drone fly (Campoy et al., 2022; Campoy, Sáez, et al., 2020). It is important to highlight that difference among sexes, populations, and/or species in body size and shape might reflect differentiation in the rate and duration of cell growth and division underlying the changes in hormonal pathways (Shingleton et al., 2007). Given that complex metabolic change at the final larval instar (Truman, 2019; Truman & Riddiford, 2019; Zhang et al., 2019) is associated with body's terminal growth period and the beginning of pupation, adult size in holometabolous insects is limited by the size of the larvae (Shingleton et al., 2007). Hence, a lack of SD in PDT in drone fly (herewith and Campoy et al., 2022) suggests that SSD obtained in both pupal and adult traits reflects developmental changes which occur during the larval stage. For instance, longer larval developmental time (LDT) in the males of *E. arbustorum* was suggested to be linked with male‐biased SSD observed in the length of second tergites in adults (Ottenheim et al., 1995). Unlike LDT, the length of PDT has been found to have a strong effect on some sexually dimorphic phenotypic traits, such as abdominal color pattern in *E. arbustorum* (Ottenheim et al., 1995). In accordance with the findings of our study, the geographic distribution of populations was shown not to have a significant effect on PDT (Ottenheim et al., 1998). To the contrary, rearing temperature has been proposed as the most important factor influencing PDT (Ottenheim et al., 1995) and phenotypic traits in eristaline adults, meaning that pupal exposure to low temperatures would cause the development of longer wings and smaller colored patches in adults (Ottenheim, 1997).

### 4.4 A consistent pattern of wing sexual shape dimorphism in drone fly

Sexual shape dimorphism was registered in WL and wing shape, the traits which are tightly linked with flight performance (e.g. Golding et al., 2001; Hedges et al., 2019; Wickman, 1992). The visualization of wing shape variation showed that differences between sexes were due to the displacement of a few landmarks, among which were those that defined the position of the r‐m vein in the central part of the wing (e.g. landmarks 7, 13, 14) and thus affected the shape of R5 cell (Appendix S8). Consequently, sex differences in wing traits in *E. tenax* were characterized by smaller and narrower wings in males and vice versa in females. Interestingly, the r‐m vain was recognized as the most variable position that caused variation in wing shape in phylogenetically divergent species (*Drosophila, Musca*, and *Ceratitis*) (Reis et al., 2021). The consistent pattern of wing SShD across populations documented herewith additionally confirmed the previous findings related to the inter‐population and trans‐generation patterns in the variation of the wing traits of drone fly (Francuski, 2012; Francuski et al., 2011; Francuski, Djurakic, Ståhls, & Milankov, 2014). Consistent SD in wing shape is evident in other hoverfly taxa, suggesting that wing shape in hoverflies is sex‐specific. For instance, females possess broader and more rounded wings in *Cheilosia* (Ludoški, 2008; Ludoški et al., 2008; Milankov et al., 2010a, 2010b) and *Merodon* species (Milankov et al., 2013), while the displacement of inner landmarks caused variation in the relative position of veins in the central part of the wings in *Eristalinus aeneus* (Ludoški, unpublished data), *Sphaerophoria scripta* (Gojković et al., 2020), and *Eristalis tenax* (Francuski, 2012; Francuski et al., 2011; Francuski, Djurakic, Ståhls, & Milankov, 2014, results herein).

The pattern of relative wing uniformity in SShD in hoverfly taxa likely represents developmental constrains in wing morphogenesis, although stabilizing selection and/or physiological constraints might be additional factors that affect its evolutionary conservatism. Indeed, both developmental and genetic constraints can limit the direction of phenotypic evolution (Pélabon et al., 2014). For instance, wing size and shape variation can be caused by differences in cell number or cell size, implying developmental basis of size and shape variation (Reis et al., 2021). On the other hand, a common pattern of SShD among closely related congeneric species, as well as diversification in the patterns and amount of sex‐based wing shape variation in distantly related hoverfly species, implied that wing shape might be driven by evolutionary canalization, as well (Bolstad et al., 2015). Indeed, evolutionary history has been hypothesized to constrain the evolution of SShD (Bolstad et al., 2015; Gidaszewski et al., 2009). Yet, the phylogenetic signal in wing morphology of hoverfly taxa remains underexplored.

### 4.5 Wing traits sexual dimorphism is related to sexually dimorphic behavior

Our study revealed that smaller males (i.e. smaller wing CS) have greater WL, which was found to be significantly different between sexes in Kikinda sample. In addition to smaller size, greater WL in males likely contributes to greater flight power and acceleration, as has been previously suggested (Wickman, 1992). Besides WL capacities, wing size, wing shape, and flight muscles affect flight performance, as well (e.g. Hedges et al., 2019). As *E. tenax* adults adhere to Batesian mimicry in the appearance (black with yellow bands across thorax and abdomen) and flight behavior of the European honey bee *Apis mellifera* L. (Golding et al., 2001), SD in wing traits results from a complex of factors. First, sex differences in the (mimetic) flight behavior (flight velocities, flight trajectories, and the percentage of time spent hovering; Golding et al., 2001) of drone fly might be caused by the differential pressure of predation (=birds) to sexes (Heal, 1979). Since drone fly males aggressively defend patches of flowers (1–2 m^2^), spending more time on hovering than females, they are particularly vulnerable to predation by birds (Golding et al., 2001; Heal, 1979). In addition, territoriality and flight behavior were found to be strongly sexually dimorphic in drone fly, especially in the case of accompanying conspecifics (Thyselius & Nordström, 2016). Therefore, Lozier et al. (2021) suggested that a trade‐off between natural and sexual selection acting on mimicry, foraging, and flight performance specified traits related to flight performance, particularly in males, and, thus, sexual segregation. Finally, the sexually dimorphic behavior in drone fly (Golding et al., 2001) is accompanied by sexually dimorphic eye design and neurophysiology (Nordström et al., 2008; Thyselius & Nordström, 2016), and sex‐biased WL, SShD, and FBSSD (results herein).

### 4.6 A consistent allometric pattern of morphological covariation

With the respect to our second goal, allometric effect on wing SShD was found to be more pronounced than in pupal body SShD. Still, the same allometric patterns of pupal body shape and wing shape variation were revealed among populations (non‐significant Population × Sex × CS and Sex × CS effects). Such consistent allometric patterns, accompanied with the conserved pattern of SShD (e.g. Francuski et al., 2011; Francuski, Djurakic, Ludoški, et al., 2014; Francuski, Djurakic, Ståhls, & Milankov, 2014; Ludoški et al., 2008; Milankov et al., 2010a, 2010b, 2013), might be developmentally (Bolstad et al., 2015; Reis et al., 2021) and/or evolutionarily constrained, suggesting species‐specific adaptations and plastic responses. For instance, the percentage of wing shape variation explained by allometry was found to range from 34% (*Drosophila melanogaster*) to 16% (*Ceratitis capitata*) and 13% (*Musca domestica*), which was congruent with species‐specific scaling relationships (Reis et al., 2021).

Herein, allometric relationships between morphometric traits (pupal: L, W, and body CS; adult: WA, WL, and wing CS) and respective pupal and adult body masses revealed the positive relationship in both sexes. Major axis regression analyses performed in the current study showed that the slope of the relationships between log‐transformed female and male mean values for pupal W differed significantly from 1 (MA slope < 1), while pupal L, L/W ratio, CS, and wing CS expressed isometric relationships. However, sex differences in plasticity have not been confirmed in the most of the explored allometric relationships of traits in the current study, although a broad phenomenon of sex‐biased sensitivity to environmental conditions and perturbations has been explained by different selective processes and evolutionary forces acting on sexes (Cox et al., 2007; Fairbairn, 2005; Székely et al., 2007). Indeed, according to the differential‐plasticity hypothesis (Fairbairn, 2005), variation in SSD among conspecific populations reflected the differential response of females and males to variable environmental factors, such as diet and temperature, factors with the greatest influence on growth rate and body size in ectothermic animals (Fox et al., 2007). Finally, our findings confirmed the notion that sex differences in sensitivity vary not only among species but also among traits within species, as has been already suggested (Rhen, 2007). Still, an extensive study of sex differences in plasticity, generated by Rensch's rule, would presumably uncover the existence of systematic patterns within the family Syrphidae.

### 4.7 Incongruent allometric scaling of morphological covariation in traits of drone fly pupae and adults

Regarding the third goal, we assessed the pattern of ontogenetic and static allometry based on allometric slopes and intercepts estimated for pupal and adult traits. First, by comparing the patterns of covariance for body mass and morphometric traits of pupae (L, W, body CS) and adults (WA, WL, wing CS), incongruence in allometric scaling was found. Greater variation in allometric slopes in adult traits showed that static allometries of the two distinct stages differed (Appendix S10). Contrary to the allometric slope, large variation of allometric intercepts was found to be a common feature of static allometry at the pupal stage (Appendix S10). Regarding the pattern of ontogenetic allometry (female vs. male CS in pupae and adults), allometric slopes were found not to differ between developmental stages, contrary to the allometric intercepts (Figure 5). Moreover, the homogeneity of allometric slopes between sexes was documented, while sex‐related changes in the intercept were found to be exclusive for adult traits (body mass vs. WA and WL) (Figure 6). Finally, the similar values of allometric scaling in geographically distinct populations analyzed in this study could result from an extensive gene exchange that prevents the evolution of locally adapted morphological covariation (see Fairbairn et al., 2007).

Given that allometric slope reflects the difference in growth rate between traits among populations or species (evolutionary allometry) and within one group of organisms (sex, ontogenetic stage, population, species) measured at same (static allometry) or different (ontogenetic allometry) developmental stages, its constancy could be explained by stabilizing selection and/or developmental/physiological constraints (allometric constraint hypothesis) (Anzai et al., 2017; Bolstad et al., 2015; Houle et al., 2019; Pélabon et al., 2014; Voje et al., 2014). More importantly, the range of studies on the allometric patterns of morphological covariation provided evidence that allometric slope harbored little genetic variation that limited the response of particular phenotypes to selection (e.g. Egset et al., 2012, see references herein, but see Houle et al., 2019), and, as such, was a potential constraint on evolutionary change (Huxley's constraint hypothesis, Huxley, 1932).

In contrast, allometric intercept (allometric elevation; the value on *y* axis where the line intercepts the vertical axis) has been proposed as the significant variable for ontogenetic and phylogenetic changes, and, therefore a valuable taxonomic indicator of speciation processes (Anzai et al., 2017). Indeed, allometric slope has been shown to be less variable across taxa, while most of the changes in static allometry were influenced by the variation of allometric intercept (Bonduriansky, 2007; Pélabon et al., 2014; Voje et al., 2014). The intercept was also found to be more evolvable due to high‐genetic variance, meaning that it can evolve faster across populations and recently diverged species (Melin et al., 2021; Rohner, 2020). Therefore, based on the findings presented herewith, the allometric intercepts are considered to have been an important source of intraspecific variation and SD in drone fly adults. However, extensive future studies of variation among conspecific populations in SSD and allometric patterns are still warranted in the Syrphidae family.

## AUTHOR CONTRIBUTIONS

**Jasmina Ludoški:** Conceptualization (equal); data curation (equal); formal analysis (lead); investigation (equal); methodology (equal); resources (equal); validation (equal); visualization (equal); writing – original draft (equal); writing – review and editing (equal). **Ljubinka Francuski:** Conceptualization (equal); data curation (equal); investigation (equal); resources (equal); writing – review and editing (equal). **Nemanja Gojković:** Data curation (equal); formal analysis (equal); investigation (equal); visualization (equal); writing – original draft (equal); writing – review and editing (equal). **Bojana Matić:** Data curation (equal); formal analysis (equal). **Vesna Milankov:** Conceptualization (equal); funding acquisition (lead); methodology (equal); project administration (lead); resources (equal); supervision (lead); validation (equal); writing – original draft (equal); writing – review and editing (equal).

## CONFLICT OF INTEREST STATEMENT

The authors declare no conflict of interest.

## Supporting information


Appendix S1–S10

## Data Availability

The DNA sequences analyzed in the manuscript have been archived in GenBank. GenBank accessions of newly uploaded sequences were given in Appendix S3.
