# Supplementary material for: Sexual size and shape dimorphism, and allometric scaling in the pupal and adult traits of Eristalis tenax
Source: Ecol Evol. 2023 Mar 15;13(3):e9907. doi: 10.1002/ece3.9907 (PMC10015363; doi:10.1002/ece3.9907)
Supplement: Supplementary file 1 — Appendix S1–S10 [file ECE3-13-e9907-s001.docx]

**Appendix S1**


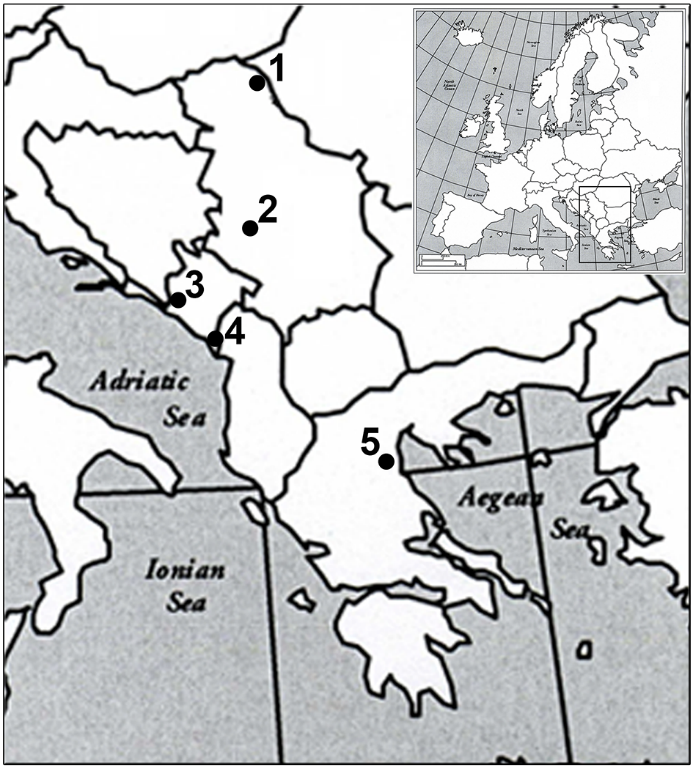


Sampling locations: 1. Kikinda, Serbia; 2) Čačak, Serbia; 3) Orjen Mt, Montenegro; 4) Šasko Lake, Montenegro; 5) Litochoro, Greece.

**Appendix S2**

| Population origin and sample size of *Eristalis tenax* used for genetic analyses. | | | | | |
| --- | --- | --- | --- | --- | --- |
| Country | Population | Longitude/Latitude | Date | Allozyme  nDNA loci | *COI* mtDNA +  *ITS2* rDNA |
| Serbia | Čačak | 20°21’E 43°53’N | 24/05/2011 | 16 | 3+3 |
| Montenegro | Orjen Mt | 18°31’E 42°28’N | 20/06/2011 | 19 | 3+2 |
|  | Šasko Lake | 19°20’E 41°58’N | 25/06/2011 | 18 | 1+2 |
| Greece | Litochoro | 22°28’E 40°06’N | 22/05/2011 | 18 | 4+4 |
|  | Total |  |  | 71 | 11+11 |

**Appendix S3**

| Details concerning *Eristalis tenax* individuals which were sequenced at *COI* mtDNA and *ITS2* rDNA loci. Shaded boxes indicate individuals previously sequenced at *COI* mtDNA locus (Francuski et al., 2014). ID – specimen ID, GAN – GenBank accession number, / - unsuccessful sequencing. | | | | | | |
| --- | --- | --- | --- | --- | --- | --- |
|  |  |  | *COI* mtDNA | | *ITS2* rDNA | |
| Country | Population | ID | GAN | Haplotype | GAN | Allele |
| Serbia | Čačak | NS686 | OM438166 | A | OM455376 | - |
|  |  | NS687 | OM438167 | A | OM455377 | + |
|  |  | NS688 | OM438168 | A | OM455378 | + |
| Montenegro | Orjen Mt | NS458 | KC763494 | A | OM455379 | + |
|  |  | NS459 | KC763495 | A | / | / |
|  |  | NS460 | KC763496 | A | OM455380 | + |
| Montenegro | Šasko Lake | NS509 | / | / | OM455381 | + |
|  |  | NS510 | OM438169 | A | OM455382 | + |
| Greece | Litochoro | NS464 | KC763498 | B | OM455383 | + |
|  |  | NS465 | KC763499 | A | OM455384 | + |
|  |  | NS466 | KC763500 | A | OM455385 | + |
|  |  | NS467 | KC763501 | A | OM455386 | + |

**Appendix S4**

**Population genetic structure analyses**

**Material and Methods**

***DNA and allozyme data***

A subset of flies, originating from four of the sampled localities (Čačak, Orjen Mt, Šasko Lake and Litochoro; Appendix 1) was used for genetic analyses: 71 individuals for the assessment of allozyme loci variability and 12 individuals for the inspection of *COI* mtDNA (655 bp) and *ITS2* rDNA (437-439 bp) polymorphism (Appendix 2). The sample included data from both the previous studies of *E. tenax* molecular variability (five allozyme loci and partial *COI* mtDNA sample) (Francuski et al., 2014; Francuski & Milankov, 2015) and the newly obtained sequences (the remaining *COI* mtDNA and *ITS2* rDNA sequences) (Appendix 3). For the newly obtained sequences, the amplification of *COI* mtDNA sequences was performed using C1-J-2183 (5′-CAA CAT TTA TTT TGA TTT TTT GG-3′) (alias JERRY) and TL2-N-3014 (5′-TCC AAT GCA CTA ATC TGC CAT ATT A-3′) (alias PAT) primers (Simon et al., 1994), while the amplification of *ITS2* rDNA sequences was performed using ITS2A (5’-TGT GAA CTG CAG GAC ACA T-3’) and ITS2B (5’-TAT GCT TAA ATT CAG GGG GT-3’) (Beebe & Saul, 1995) primers, under identical PCR conditions. The success of amplification reactions was verified by running PCR products on a 1.5% agarose gel stained with ethidium bromide and follow-up visualization under UV light. Upon the confirmation of their successful amplification, the products were purified using ExoSAP-IT™ PCR Product Cleanup Reagent (Thermo Fisher Scientific, Vilnius, Lithuania), and bidirectionally sequenced by Macrogen (the Netherlands) on ABI3730XL using the same sets of primers used for the PCRs. Chromatograms retrieved by sequencing were inspected in Chromas 2.6 (Tehnelysiumm Pty Ltd) for erroneously called bases, edited, and aligned together with the previously published sequences in BioEdit 7.0.5.3 (Hall, 1999) using ClustalW algorithm (Thompson et al.,1994) to obtain final data sets. In total, 11 *COI* mtDNA sequences (seven previously published and four successfully sequenced in this study) and 11 *ITS2* rDNA sequences (successfully *de novo* sequenced) were analyzed (Appendix 3). The newly obtained sequences were uploaded to GenBank (Appendix 3).

***Statistical analyses***

*Eristalis tenax* population genetic structure was assessed solely using five allozyme loci due to the lack of the variability of *COI* mtDNA and *ITS2* rDNA loci (see Results). The analyses were conducted using two distinct Bayesian clustering approaches: non-spatial, individual-based approach implemented in STRUCTURE 2.3.4 (Pritchard et al., 2000) and spatial, individual-based approach implemented in BAPS 6 (Corander & Marttinen, 2006; Corander et al., 2008). STRUCTURE analysis used an admixture ancestry model and correlated allele frequency model. Ten independent replicates for each value of *K* (number of genetic clusters) were ran, with *K* varying from one to four (corresponding to four geographic samples). Every run consisted of 500,000 burn-in steps, followed by 500,000 iterations. The most probable value of *K* was retrieved from CLUMPAK 1.1 (Kopelman et al., 2015) using the method of Evanno et al. (2005). Additionally, the visual representation of STRUCTURE analysis output was also retrieved using CLUMPAK, while the mean population values of cluster membership coefficients (*q*) were obtained from STRUCTURE HARVESTER 0.6 (Earl & vonHoldt, 2012) as averaged values over ten runs for the most probable value of *K*. BAPS analysis inspected the population structure by firstly implementing Spatial clustering of individuals mixture analysis and assuming *K* which varied from one to four. For each value of *K*, ten independent replicates were ran. The optimal *K* was selected based on the number of clusters for which the log of marginal likelihood was maximal. The mixture analysis was followed by admixture analysis using Admixture based on Mixture results option. The number of iterations was set to 100,000, the number of reference individuals per population to 200, and the number of iterations per reference individual to 20.

**Results**

***Population genetic structure analyses***

*Eristalis tenax* population genetic structure analysis based on allozyme data of the four geographic samples indicated *K*=2 and *K*=3 as the most probable numbers of distinct genetic clusters using non-spatial STRUCTURE and spatial BAPS analysis, respectively. However, regardless of the differences in optimal *K* estimation, both analysis types indicated a high degree of population admixture characterizing the four samples (Appendix 5, Appendix 6). Namely, apart from Orjen Mt sample in STRUCTURE analysis and Čačak sample in BAPS analysis, respective average cluster membership coefficient values were lower than 0.800 (Appendix 5).

The assessment of sequence data suggested a genetically homogenous sample, therefore no further genetic analyses were performed. Namely, two *COI* mtDNA haplotypes were retrieved, with 10/11 sequences representing an identical haplotype (haplotype A) which differed from the sole alternative representative (haplotype B; NS464 sampled in Litochoro, Greece) by a single substitution (Appendix 3). Similarly, two *ITS2* rDNA haplotypes were retrieved, with 10/11 sequences representing an identical allele (allele +) which differed from the alternative representative (allele ˗; NS686 sampled in Čačak, Serbia) by a single two-base indel (Appendix 3).

**References**

Beebe, N.W., & Saul, A. (1995). Discrimination of all members of the *Anopheles punctulatus* complex by polymerase chain reaction-restriction fragment length polymorphism analysis. *American Journal of Tropical Medicine and Hygiene, 53*, 478-481*.*

Corander, J., & Marttinen, P. (2006). Bayesian identification of admixture events using multi-locus molecular markers. *Molecular Ecology*, *15*(10), 2833-2843.

Corander, J., Sirén, J., & Arjas, E. (2008). Bayesian spatial modelling of genetic population structure. Comput. *Computational Statistics,* *23*, 111-129.

Earl, D.A., & vonHoldt, B.M. (2012). STRUCTURE HARVESTER: A website and program for visualizing STRUCTURE output and implementing the Evanno method. *Conservation Genetics Resources*, *4,* 359-361.

Evanno, G., Regnaut, S., & Goudet, J. (2005). Detecting the number of clusters of individuals using the software structure: A simulation study. *Molecular Ecology,* *14*(8), 2611-2620.

Francuski, Lj., & Milankov, V. (2015). Assessing spatial population structure and heterogeneity in the dronefly. *Journal of Zoology,* *297,* 286-300.

Francuski, Lj., Djurakic, M., Ståhls, G., & Milankov, V. (2014). Landscape genetics and wing morphometrics show a lack of structuring across island and coastal populations of the dronefly in the Mediterranean. *Journal of Zoology,* *292,* 156-169.

Hall, T.A. (1999). BioEdit: A user-friendly biological sequence alignment editor and analysis program for Windows 95/98/NT. *Nucleic Acids Research,* *41,* 95-98.

Kopelman, N.M., Mayzel, J., Jakobsson, M., Rosenberg, N.A., & Mayrose, I. (2015). Clumpak : A program for identifying clustering modes and packaging population structure inferences across K. *Molecular Ecology Resources,* *15*(5), 1179-1191.

Pritchard, J.K., Stephens, M., & Donnelly, P. (2000). Inference of population structure using multilocus genotype data. *Genetics,* *155*(2), 945-959.

Simon, C., Frati, F., Beckenbach, A., Crespi, B., Liu, H., & Flook, P. (1994). Evolution, weighting, and phylogenetic utility of mitochondrial gene sequences and a compilation of conserved polymerase chain reaction primers. *Annals of the Entomological Society of America*, *87,* 651-701.

Thompson, J.D., Higgins, D.G., & Gibson, T.J (1994). CLUSTAL W: improving the sensitivity of progressive multiple sequence alignment through sequence weighting, position-specific gap penalties and weight matrix choice. *Nucleic Acids Research,* *22,* 4673-4680.

**Appendix S5**

| The results of population genetic structure analyses implemented in STRUCTURE (non-spatial approach) and BAPS (spatial approach) programs and based on genetic differences in allozyme loci among analyzed *Eristalis tenax* individuals. The presented mean values of the cluster membership coefficients (*q*) of geographic samples to the retrieved genetic clusters were summarized over ten runs in STRUCTURE, while average population admixture coefficients were retrieved in BAPS. The shaded boxes indicate highly genetically differentiated geographic samples (average sample coefficient values higher than 0.800). | | | |  |
| --- | --- | --- | --- | --- |
| **STRUCTURE, *K*=2** | ***K1*** | ***K2*** | ***K3*** | |
| Čačak | 0.708 | 0.292 |  | |
| Orjen Mt | 0.130 | 0.870 |  | |
| Šasko Lake | 0.432 | 0.568 |  | |
| Litochoro | 0.474 | 0.526 |  | |
| **BAPS, *K*=3** |  |  |  | |
| Čačak | 0.125 | 0.875 | 0.000 | |
| Orjen Mt | 0.421 | 0.053 | 0.526 | |
| Šasko Lake | 0.556 | 0.444 | 0.000 | |
| Litochoro | 0.500 | 0.444 | 0.056 | |

**Appendix S6**


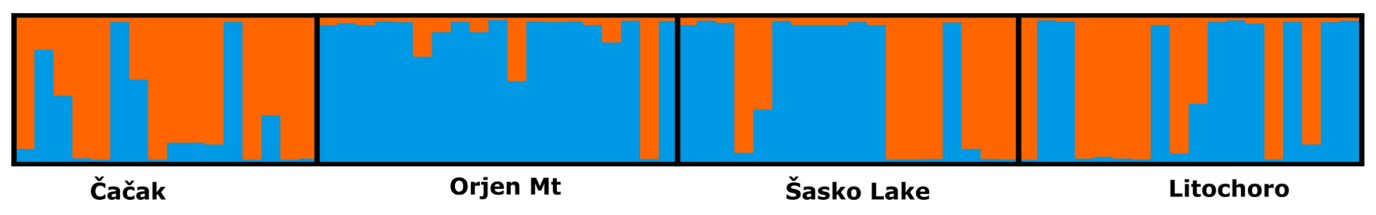


The visual representation of the STRUCTURE population genetic structure analysis of *Eristalis tenax* geographic samples based on allozyme loci.

**Appendix S7**

| Sexual size dimorphism in *Eristalis tenax*. Descriptive statistics include the mean size and standard deviation of traits (Mean ± SD) and sexual size ratio (SDI; after Lovich & Gibbons, 1992) for pupal body length (L), width (W), length/width ratio (L/W) and centroid size (pupa CS), and adult wing centroid size (adult CS). Significance for all measurements was calculated with *t*-test (ns - not significant, ** p < 0.01, *** p < 0.001). Major axis slope (MA slope) with lower and upper values of 95% confidence interval (95% CI) and intercept (*a*) were obtained by linear regression of sex trait mean size. | | | | | | |
| --- | --- | --- | --- | --- | --- | --- |
|  | Female | Male | *t*-test | SDI | MA slope | *a* |
|  | Mean ± SD | Mean ± SD |  |  | (95% CI) |  |
| **L** |  |  |  |  |  |  |
| Kikinda | 13.65 ± 0.44 | 13.52 ± 0.54 | ns | 0.010 | 0.568 | 0.488 |
| Čačak | 13.84 ± 0.88 | 13.74 ± 0.94 | ns | 0.007 | (0.373, 2.764) |  |
| Orjen Mt | 13.72 ± 0.70 | 13.49 ± 0.70 | ns | 0.017 |  |  |
| Šasko Lake | 12.83 ± 0.80 | 12.20 ± 1.17 | ns | 0.052 |  |  |
| Litochoro | 12.69 ± 0.53 | 12.22 ± 0.42 | ** | 0.038 |  |  |
| **W** |  |  |  |  |  |  |
| Kikinda | 5.79 ± 0.26 | 5.62 ± 0.28 | ns | 0.030 | 0.670 | 0.256 |
| Čačak | 5.63 ± 0.48 | 5.61 ± 0.50 | ns | 0.004 | (-0.919, 0.892) |  |
| Orjen Mt | 5.81 ± 0.36 | 5.72 ± 0.20 | ns | 0.016 |  |  |
| Šasko Lake | 5.44 ± 0.45 | 5.18 ± 0.53 | ns | 0.050 |  |  |
| Litochoro | 5.41 ± 0.23 | 5.15 ± 0.26 | ** | 0.050 |  |  |
| **L/W** |  |  |  |  |  |  |
| Kikinda | 2.36 ± 0.11 | 2.41 ± 0.10 | ns | -0.021 | 1.167 | -0.065 |
| Čačak | 2.47 ± 0.14 | 2.46 ± 0.15 | ns | 0.004 | (0.624, 3.341) |  |
| Orjen Mt | 2.37 ± 0.12 | 2.36 ± 0.10 | ns | 0.004 |  |  |
| Šasko Lake | 2.37 ± 0.12 | 2.36 ± 0.15 | ns | 0.004 |  |  |
| Litochoro | 2.35 ± 0.12 | 2.38 ± 0.12 | ns | -0.013 |  |  |
| **Pupa CS** |  |  |  |  |  |  |
| Kikinda | 2388.82 ± 74.59 | 2354.60 ± 84.07 | ns | 0.015 | 0.686 | 1.063 |
| Čačak | 2392.25 ± 153.61 | 2377.31 ± 163.98 | ns | 0.006 | (0.030, 1.223) |  |
| Orjen Mt | 2408.19 ± 114.87 | 2368.81 ± 107.40 | ns | 0.017 |  |  |
| Šasko Lake | 2255.43 ±137.30 | 2147.38 ± 202.26 | ns | 0.050 |  |  |
| Litochoro | 2216.58 ± 82.85 | 2127.68 ± 65.87 | ** | 0.042 |  |  |
| **Adult CS** |  |  |  |  |  |  |
| Kikinda | 1824.15 ± 41.82 | 1674.13 ± 60.14 | *** | 0.090 | 0.608 | 1.300 |
| Čačak | 1835.72 ± 81.43 | 1704.52 ± 80.03 | *** | 0.077 | (0.208, 1.067) |  |
| Orjen Mt | 1831.08 ± 73.20 | 1719.48 ± 56.85 | *** | 0.065 |  |  |
| Šasko Lake | 1764.23 ± 73.94 | 1619.54 ± 92.74 | *** | 0.089 |  |  |
| Litochoro | 1792.75 ± 48.93 | 1627.38 ± 52.86 | *** | 0.102 |  |  |

**Appendix S8**


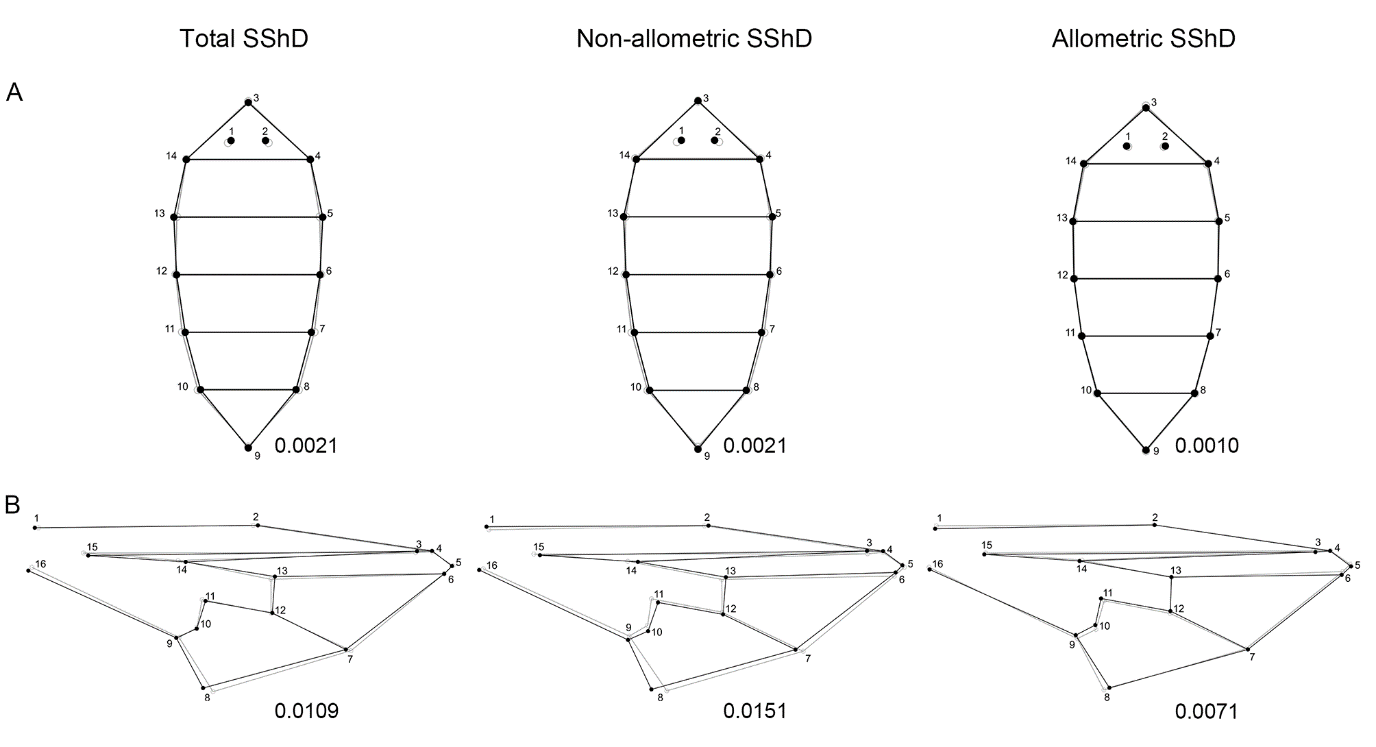


The visualization of the pupa body (A) and adult wing (B) shape variation of *Eristalis tenax*. Shape changes (black lines and solid circles) from the consensus configuration of landmarks (gray lines and empty circles) associated with variation along the discriminant axis in positive direction is presented. The amount of total SShD and its non-allometric and allometric components are given in the units of Procrustes distance. Numbers in the wireframe graph refer to landmarks shown in Figure 2.

**Appendix S9**

| The multivariate regression of shape on centroid size of *Eristalis tenax* testing for allometry. Results represent the percentage of total shape variation explained by size. | | | | | | |
| --- | --- | --- | --- | --- | --- | --- |
|  | **Pupal body shape** | | | **Wing shape** | | |
|  | % explained | | | % explained | | |
|  | All | Female | Male | All | female | Male |
| Kikinda | 1.54^ns^ | 3.32 ^ns^ | 2.62 ^ns^ | 1.67 ^ns^ | 8.38 ^ns^ | 6.07 ^ns^ |
| Čačak | 3.60* | 4.79 ^ns^ | 3.67 ^ns^ | 7.02*** | 5.68* | 10.17*** |
| Orjen Mt | 3.94 ^ns^ | 0.68 ^ns^ | 21.98*** | 5.02** | 4.69 ^ns^ | 13.26** |
| Šasko Lake | 0.67 ^ns^ | 4.09 ^ns^ | 0.83 ^ns^ | 22.37*** | 18.28* | 30.60*** |
| Litochoro | 4.77 ^ns^ | 19.03 ^ns^ | 3.47 ^ns^ | 4.77 ^ns^ | 7.43 ^ns^ | 6.11 ^ns^ |
| ns - not significant; * p < 0.05; ** p < 0.01; *** p < 0.001 | | | | | | |

**Appendix S10**

| The linear regression of *Eristalis tenax* pupal body mass to pupal body length (L), width (W) and centroid size (CS), as well as adult body mass to wing area (WA), wing loading (WL) and centroid size (CS). The regression line slopes (MA slopes) obtained for two sexes were compared with chi^2^ test (ns - not significant). *a* - intercept; 95% CI- lower and upper values of 95% confidence interval. | | | | | |
| --- | --- | --- | --- | --- | --- |
|  | Female | | Male | |  |
|  | MA slope (95% CI) | *a* | MA slope (95% CI) | *a* | Chi^2^ |
| **L** |  |  |  |  |  |
| Kikinda | 0.187 (-0.060, 0.401) | 0.720 | 0.263 (0.095, 0.494) | 0.554 | ns |
| Čačak | 0.176 (-0.071, 0.368) | 0.759 | 0.290 (0.165, 0.416) | 0.499 | ns |
| **W** |  |  |  |  |  |
| Kikinda | 0.398 (0.288, 0.558) | -0.120 | 0.387 (0.305, 0.590) | -0.101 | ns |
| Čačak | 0.369 (0.216, 0.598) | -0.067 | 0.343 (0.217, 0.458) | -0.007 | ns |
| **Pupa CS** |  |  |  |  |  |
| Kikinda | 0.265 (0.112, 0.404) | 2.789 | 0.262 (0.193, 0.417) | 2.796 | ns |
| Čačak | 0.212 (0.092, 0.326) | 2.914 | 0.291 (0.213, 0.374) | 2.735 | ns |
| **WA** |  |  |  |  |  |
| Kikinda | 0.336 (0.131, 0.462) | 0.834 | 0.482 (0.317, 1.442) | 0.494 | ns |
| Čačak | 0.056 (-0.119, 0.151) | 1.419 | 0.194 (0.061, 0.303) | 1.083 | ns |
| **WL** |  |  |  |  |  |
| Kikinda | 0.704 (0.603, 0.908) | -1.215 | 0.689 (-0.080, 0.950) | -1.126 | ns |
| Čačak | 0.969 (0.880, 1.119) | -1.773 | 0.868 (0.705, 0948) | -1.507 | ns |
| **Adult CS** |  |  |  |  |  |
| Kikinda | 0.145 (-0.495, 0.247) | 3.093 | 0.199 (0.090, 0.590) | 2.987 | ns |
| Čačak | 0.118 (0.031, 0.208) | 3.161 | 0.141 (-0.002, 0.216) | 3.144 | ns |
